# Adventure Legal Medicine: a free online serious game for supplementary use in undergraduate medical education

**DOI:** 10.1007/s00414-023-02946-x

**Published:** 2023-01-10

**Authors:** Sven Anders, Antonia Steen, Tjark Müller, Waldemar Krause, Annika Sanwald, Tobias Raupach, Benjamin Ondruschka, Oliver Krebs

**Affiliations:** 1 Institute of Legal Medicine, University Medical Center Hamburg-Eppendorf, 22529 Hamburg, Germany; 2 Leibniz-Institut Für Wissensmedien, Tübingen, Germany; 3 Department of Medical Education, University Hospital Bonn, Bonn, Germany

**Keywords:** Legal medicine, Serious game, Medical education, Computer-based learning

## Abstract

Serious games (computer-based learning games) are increasingly used in medical education at various levels, as user access is independent of location and time and promotes non-linear learning. In legal medicine, interactive digital media are still scarce. The freely accessible online serious game “Adventure Legal Medicine” was developed as part of the “Hamburg Open Online University”. The goal was to teach the basics of forensic casework in a point-and-click adventure setting consisting of five cases. During development, 40 medical students were asked to evaluate the game anonymously. The System Usability Scale (SUS) resulted in a mean score of 86.7 (SD 8.3), which corresponds to above-average usability. Further specific evaluations revealed a good to very good rating of the game with no differences in terms of gender (*p* = 0.214), first-year versus advanced students (*p* = 0.393) and students who never/rarely or sometimes/often played computer games (*p* = 0.780). Since there are only a few digital media so far that allow curricular integration into undergraduate teaching in legal medicine, this serious game represents a possibility to integrate digital media into both face-to-face teaching and distance learning and to use it as a supplement to the medical school’s own teaching offer, encouraging users to actively engage with the subject.

## Introduction

Serious games (i.e. computer-based learning games, primarily focused on education rather than entertainment) are increasingly used in education at various levels. In medicine, serious games are used in patient education, as programmes for training skills and for learning and transferring knowledge in undergraduate medical education [1–3].

In the field of legal medicine, there are hardly any online teaching resources so far that present knowledge content or offer the possibility to recapitulate teaching content. In 2011, Schmeling et al. presented a game-based approach to case-based learning and practice of external post-mortem examination [4], which can be used in both undergraduate and postgraduate training. This is an institutional or fee-based service and thus not freely accessible, so widespread use is not readily possible. In contrast, ease of accessibility and low cost are thought to support the use of online teaching resources [3].

In addition to the advantages of location- and time-independent access for users, game-based learning offers the possibility of non-linear learning, which is only possible to a limited extent in traditional teaching formats [5] or for the integration of voluntary additional offers that go beyond the compulsory curriculum of medical training [6].

While the perception of serious games on the user side is generally very good and such resources aim at increasing learner engagement, motivation and retention by fostering intrinsic motivation of individual users (self-determination theory [7]), there are no consistent results on the benefits of serious games per se in terms of learning success [3]. However, it has been shown that when used in a targeted manner, knowledge transfer can be promoted [8] and learning success can be superior to that of face-to-face teaching [9].

We report on the development, evaluation and usability of a freely available online serious game that deals with the basics of various aspects of legal medicine.

## Material and methods

The serious game “Adventure Legal Medicine” was developed and financially supported within the framework of the “Hamburg Open Online University” (HOOU). HOOU is a project in which educational materials are developed as Open Education Resources (OEM) and made available online free of charge and barrier-free. Use is not limited to students in the respective departments, but is also extended to the interested public (www.hoou.de).

The game was developed and implemented by an interdisciplinary working group consisting of a forensic pathologist, a forensic anthropologist, a psychologist, a medical student, a game designer and a graphic artist.

**Fig. 1:**
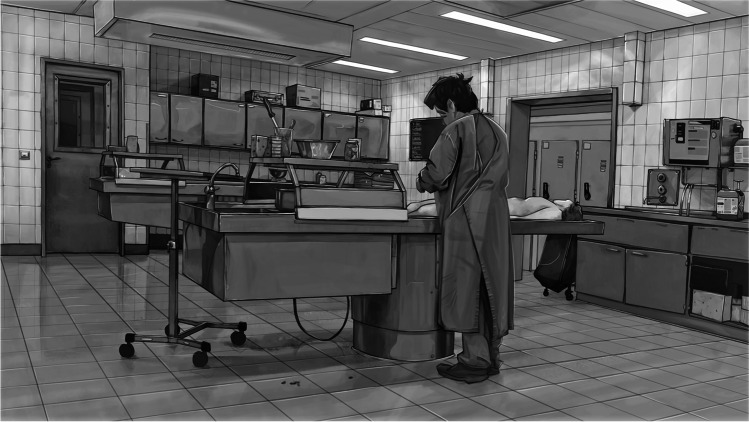
Exemplary impression of the black and white graphic novel-style graphics of the game

The aim was to convey the basics of forensic medical work in a case-oriented point-and-click adventure setting. The users are given specific tasks based on a case, which they have to solve in the course of the game. At the end of each case, the users receive feedback from a virtual senior physician. Throughout the game, the users have access to a short digital textbook explaining the methods and background presented, providing texts which were also developed as part of the conception of the game. In line with the OEM concept of HOOU, the target groups consist of medical students on the one hand, who can use the game to self-test their knowledge of the topics covered in the curricular teaching, and the interested public on the other hand. It should be emphasized that the cases and accompanying texts are merely introductory and didactic. The game cannot and does not intend to represent a critical discussion of the methods in the sense of a more advanced specialized literature. The broad target group had to be taken into account in the selection of topics, the type of presentation and linguistic aspects. To ensure a neutral visual presentation, the graphics were kept in a black and white graphic novel style (Fig. 1). The programming was done with the game engine Unity (Unity Technologies, San Francisco, USA).

The selection of topics should cover a spectrum as broad as possible. After interdisciplinary discussion in the development team, the following topics were selected: (i) time of death estimation, (ii) post-mortem external examination, (iii) forensic anthropology, (iv) DNA analysis/paternity test, (v) autopsy/alcohol and drug influence.

Below is a brief description of the content of the different cases. During the cases, the user is assisted by the integrated textbook, and feedback on the results obtained is given at the end of each case.

(i) Time of death estimation: A person is found in a flat. The user has to collect different parameters for the time of death diagnosis with the corresponding tools and link the individual examination results. Subsequently, an estimation of the time since death has to be made.
(ii) External post-mortem examination: A person is found on the bank of a river. The user has to undress the person and examine the different body regions. This results in several findings from which it must be assessed whether there is evidence of a non-natural manner of death.
(iii) Forensic anthropology: Several bones of a human skeleton are found in a forest. After taking measurements of the bones, estimates of age, sex and height are to be made and a conclusion is to be drawn from “missing person” data sheets as to whether the bones could belong to one of these individuals.
(iv) DNA analysis/paternity test: The person from case (ii) has been identified to be a wealthy recluse and four people came forward claiming to be heirs. After the steps of a DNA analysis have been schematically traced, the DNA patterns of the deceased and the four persons are to be used to identify the biological child.
(v) Autopsy/alcohol and drug influence: A person is recovered from an accident vehicle. First, an external inspection is carried out, then the organs are to be examined step by step. In addition, a toxicological analysis is carried out. Finally, the findings are to be interpreted.

After the development and programming of a first version of cases 1 and 2 (pre-test version), five volunteers were asked to pilot-test this version without external guidance (2 males, 25 and 49 years old; 3 females, 21, 25 and 54 years old) and to express during the game, in the sense of a think-aloud, what thoughts they had about the game controls while performing each step. These statements were recorded by an independent observer. In addition, the subjects were asked to complete a validated, system-independent questionnaire on the usability of the game (System Usability Scale, SUS) [9]. The SUS consists of 10 items that are rated on a 5-point Likert scale. A weighted score from 0 to 100 is calculated from the scale values. A score above 68 is considered above average [11].

After adapting individual game steps of cases 1 and 2, taking into account the comments of the five subjects, case 3 was created and programmed bearing in mind the comments of the five inspectors on the gameplay.

Forty medical students were asked to test the adapted version of the game with cases 1, 2 and 3 (test version). As the game was also to be made available to the interested public, students with different levels of education were asked to volunteer to participate in the testing anonymously. Students were then asked to complete the SUS questionnaire and an additional questionnaire with 16 items (6-point Likert scale, 1 = strongly agree, 6 = strongly disagree) specifically related to the game in question (Table 1). In addition, there was an opportunity to provide free comments on the gameplay.

**Table 1:** Results of the 16-item questionnaire (6-point Likert scale) specifically related to the game (*n* = 40)

Item	Mean	SD (95%)
(1) The start page of the game appealed to me	2.40	1.172
(2) The game was easy to understand and intuitive to use	1.70	0.758
(3) I understood the information I collected with the help of the tools in the game	1.48	0.679
(4) I understood how to apply the tools for the investigations “in reality”	1.98	0.920
(5) Sometimes I didn’t know what was being asked of me because I didn’t understand the game management	3.73	1.601
(6) The graphics of the game appealed to me very much	1.90	0.928
(7) The music and the acoustic signals have supported the game	2.23	1.266
(8) The feedback at the end of case 1 was detailed enough	1.78	1.149
(9) The feedback at the end of case 2 was detailed enough	1.61	1.022
(10) The feedback at the end of case 3 was detailed enough	2.22	1.396
(11) Overall, I enjoyed the game	1.70	0.740
(12) I have gained a better idea of how the time of death is narrowed down (case 1)	1.47	0.706
(13) I have gained a better idea of what is looked for in an postmortem external examination (case 2)	1.58	0.732
(14) I have gained a better idea of how to examine bone finds (case 3)	1.69	0.786
(15) I have a better overview of the work in forensic medicine	1.73	0.902
(16) I would give the game the following school grade (German school grade system, 1 = very good, 6 = poor)	1.84	0.602
“Overall impression” score (inverted sum score of items 12–16; 0–25 points)	21.88	2.941

Informed consent was given by all participants. According to the guidelines of the responsible ethics committee, no approval was required.

Since an influence of gender, medical knowledge and frequency of use of computer games can be hypothesized, group comparisons (*t*-test) were conducted using IBM SPSS Statistics 24.0 (IBM Corp, Armonk, IBM SPSS Statistics 24.0 (IBM Corp, Armonk, USA).

Subsequently, cases 4 and 5 were developed, graphically designed and programmed.

The complete, final version of the game with cases 1 to 5 is freely available online at http://elearning.uke.de/HOOU/RechtsmedizinSeriousGame/, via the HOOU site (www.hoou.de) and in a version for mobile devices (mobile phone or tablet, Android operating systems) via the Google Play Store under the German name “Abenteuer Rechtsmedizin”. On the start page, a selection can be made between German and English language.

## Results

The mean SUS score of the 5 subjects who had played the pre-test version with cases 1 and 2 was 63.5 (SD 14.3). The comments referred in particular to linguistic inaccuracies, lack of clarity in the menu, gameplay and game guidance, and the suggested colour highlighting of relevant image content during a mouseover.

In the test version (cases 1, 2 and 3), 38 of the 40 test persons completed the SUS questionnaire. This resulted in an average SUS score of 86.7 (SD 8.3). The subjects were between 17 and 34 years old (mean 21.9, SD 4.1); 26 of the subjects were female (65%) and 14 were male (35%). Of the 40 subjects, 36 provided information on the year of study (first year *n* = 22; 55%, second year *n* = 10; 25%, fifth year *n* = 4; 10%). Of the respondents, 32.5% (*n* = 13) indicated that they never play computer games, 35.0% (*n* = 14) rarely, 22.5% (*n* = 9) sometimes and 10% (*n* = 4) often.

In terms of ease of use, the SUS score showed a significant improvement between the pre-test and test versions (63.5 vs. 86.7; *p* < 0.001).

The results of the additional questionnaire completed in the test group are shown in Table 1. In addition, items 12 to 16 were inverted for a “total score” (0–5 points) and added up so that a calculated total satisfaction score of 0–25 points was conceivable (mean 21.9, SD 2.9; see Table 1). Overall, the evaluation resulted in a good to very good rating of the game. Of the 40 test persons, 33 used the free-text comment field for suggestions on comprehensibility and gameplay as well as for positive comments, which mainly referred to the game idea, the graphic implementation and the integrated textbook.

The results do not indicate a significant gender difference in overall satisfaction (*p* = 0.214; female mean 22.4, SD 2.5; male mean 21.1, SD 3.5) and no significant difference between first-year and advanced students (*p* = 0.393; first-year 21.3, SD 2.9; second- and fifth-year students 22.2, SD 2.9). Similarly, there was no significant difference between students who never/rarely or sometimes/frequently played computer games (*p* = 0.780; 21.8, SD 3.3 and 22.1, SD 2.0, respectively).

## Discussion

Due to its structure and features, the digital serious game “Adventure Legal Medicine” appears suitable for learning the basic principles of time-of-death diagnostics, post-mortem external examination, forensic anthropology, DNA and paternity diagnostics as well as autopsy including laboratory chemical post-mortem examinations without (relevant) prior knowledge. In addition, the game provides access to learning texts that are available while playing. In the context of undergraduate medical education, the game can be used as a supplement or catch up to lectures and courses. It can be used both to prepare the content of the course and to check students’ understanding of the learning content by self-testing and pointing them to cases that fit the curriculum content.

The free availability both as an online version (usable on all devices) and as a free app version (tablets and mobile phones with Android) and the availability of the game including the textbook in German and English enable a location-independent and worldwide use of the game. Further translations to other world languages are basically possible. Curricular integration is thus possible wherever legal medicine is taught to students of medicine or related disciplines such as criminology or law.

The test version with 3 cases showed above-average usability and good to very good evaluation results (Table 1). Evaluation results revealed no gender differences or indications that prior knowledge of computer games or medical knowledge was necessary for the application. The active involvement of the user, the integrated feedback and the textbook support active engagement with the content. The game was extended to 5 cases and the game guidance was further improved. Due to the open terms of use of the game, it is difficult to get feedback from users. At the time of writing this manuscript, the Android version has been downloaded more than 3000 times via the Google Play Store since the upload in January 2020 and has received an average rating of 4.5 out of 5 from the users (https://play.google.com/store/apps/details?id=com.UKE.AbenteuerRechtsmedizin&gl=DE). Unfortunately, there is no information available on how often the online version was accessed.

Since there are only minute digital offerings to date that enable curricular integration into undergraduate teaching in legal medicine, the serious adventure game presented here represents a timely and easily accessible opportunity to integrate digital media into both classroom teaching and distance learning and to use it as a supplement to the medical school’s own teaching offerings literary all over the world, encouraging users to actively engage with the subject. There is evidence from other areas of teaching in legal medicine that an active, hands-on role for students leads to greater long-term impact [12], so incorporating digital media in which learners are given an active role may be a popular way to enhance the desired learning effects.

## Data Availability

The datasets generated during and/or analysed during the current study are available from the corresponding author on reasonable request.
